# Integrating Single-Cell Transcriptome and Network Analysis to Characterize the Therapeutic Response of Chronic Myeloid Leukemia

**DOI:** 10.3390/ijms232214335

**Published:** 2022-11-18

**Authors:** Jialu Ma, Nathan Pettit, John Talburt, Shanzhi Wang, Sherman M. Weissman, Mary Qu Yang

**Affiliations:** 1 MidSouth Bioinformatics Center and Joint Bioinformatics Graduate Program, University of Arkansas at Little Rock, University of Arkansas for Medical Sciences, Little Rock, AR 72204, USA; 2 Department of Information Science, University of Arkansas at Little Rock, Little Rock, AR 72204, USA; 3 Department of Philosophy and Interdisciplinary Studies, University of Arkansas at Little Rock, Little Rock, AR 72204, USA; 4 Department of Pharmaceutical Sciences, St. John’s University, Queens, NY 11439, USA; 5 Department of Genetics, Yale School of Medicine, New Haven, CT 06520, USA

**Keywords:** single cell, RNA sequencing, chronic myeloid leukemia, network analysis, TKI responses, BCR-ABL

## Abstract

Chronic myeloid leukemia (CML) is a myeloproliferative disease characterized by a unique BCR-ABL fusion gene. Tyrosine kinase inhibitors (TKIs) were developed to target the BCR-ABL oncoprotein, inhibiting its abnormal kinase activity. TKI treatments have significantly improved CML patient outcomes. However, the patients can develop drug resistance and relapse after therapy discontinues largely due to intratumor heterogeneity. It is critical to understand the differences in therapeutic responses among subpopulations of cells. Single-cell RNA sequencing measures the transcriptome of individual cells, allowing us to differentiate and analyze individual cell populations. Here, we integrated a single-cell RNA sequencing profile of CML stem cells and network analysis to decipher the mechanisms of distinct TKI responses. Compared to normal hematopoietic stem cells, a set of genes that were concordantly differentially expressed in various types of stem cells of CML patients was revealed. Further transcription regulatory network analysis found that most of these genes were directly controlled by one or more transcript factors and the genes have more regulators in the cells of the patients who responded to the treatment. The molecular markers including a known drug-resistance gene and novel gene signatures for treatment response were also identified. Moreover, we combined protein–protein interaction network construction with a cancer drug database and uncovered the drugs that target the marker genes directly or indirectly via the protein interactions. The gene signatures and their interacted proteins identified by this work can be used for treatment response prediction and lead to new strategies for drug resistance monitoring and prevention. Our single-cell-based findings offered novel insights into the mechanisms underlying the therapeutic response of CML.

## 1. Introduction

Chronic myeloid leukemia (CML) is a type of cancer that starts in certain blood-forming cells of the bone marrow. It is reported that the prevalence of CML has tripled from 3.9 to 11.9 per 100,000 population from 1985 to 2012 and is expected to further rise to 22 per 100,000 population by 2060 [1]. The critical genetic event in CML is the formation of a BCR-ABL fusion gene through a chromosomal translocation between chromosomes 9 and 22, which is found in more than 90% of CML patients [2]. The protein product of this fusion gene causes uncontrolled CML cell growth. The BCR-ABL protein is responsible for abnormalities of the chronic phase CML (CP-CML) and is critical for the malignant transformation of CML [3]. Based on the clinical characteristics, CML has three phases: chronic, accelerated, and blast crisis. Over 85% of patients are diagnosed at the chronic phase [4]. Without treatment, CP-CML will inevitably develop into an advanced and aggressive phase. Currently, it is very difficult to treat blast crisis [5].

The introduction of tyrosine kinase inhibitor (TKI) revolutionized CP-CML therapy. TKIs target the BCR-ABL fusion protein and inhibit its tyrosine kinase activity. The seven-year survival rate of chronic phase CML patients was 90% [6] and the estimated overall ten-year survival rate of the patients with the imatinib treatment was 83.3% [7]. Many of them have a nearly normal life expectancy. However, some CML patients develop drug resistance and relapse after initial treatment [8]. Roughly one-quarter of CML patients developed TKI resistance at some time point during the treatment [9]. It has been found that the point mutations at the ATP-binding site of the BCR-ABL kinase domain decrease drug binding affinity and thus cause TKI drug resistance [8,10]. However, some patients without such mutations still respond poorly or have lost response to TKIs, suggesting heterogeneity mechanisms responsible for drug resistance.

Cancer stem cells (CSCs) represent a rare subpopulation of cancer cells. CSCs share similar characteristics to normal stem cells such as self-renewal and differentiation, as well as the ability for tumorigenesis. The substantial evidence suggested that CSCs are responsible for tumor propagation [11,12,13]. A subset of CSCs is resistant to therapy and persistent during remission, causing cancer recurrence and metastasis [14,15,16]. The progression of chronic-phase CML is driven by rare CML stem cells (CML-SCs) [17]. The CML-SCs show selective tolerance to TKI treatment [18,19]. The residuals of CML-SCs exist in most patients, resulting in relapse after therapy discontinuation [18,19,20]. Additionally, several recent studies utilizing mouse models suggested that non-clonal BCR-ABL^−^ stem cells were involved in the CML disease phenotype [21,22]. It remains to be elucidated how disruption of the BCR-ABL^−^ stem cells (non-leukemic hematopoietic stem cells) might be associated with the therapeutic response [23,24]. Giustacchini et al. developed a BCR-ABL-target Smart-seq2 protocol for high sensitive BCR-ABL detection. The technology can detect distinct gene expression in BCR-ABL^+^ and BCR-ABL^−^ stem cells within the same CML patient. They found that CP-CML patients having stem cells with quiescent properties were more likely to develop TKI resistance [23].

In this study, we developed a computational approach combining the analysis of single-cell RNA sequencing (scRNA-seq) of CML-SCs [23] and network inference to identify gene markers as well as their transcription regulators that are associated with TKI response. Four groups of stem cells, based on the TKI response (good vs. poor) of patients and BCR-ABL status (positive vs. negative), were analyzed. Differential expression analysis uncovered a set of common genes that were differentially expressed in the four stem cell groups as compared to normal hematopoietic stem cells (HSCs). To identify regulators of these genes, we constructed the transcription regulatory networks by integrating multi-layer genomic information including gene co-expression module, transcription factor (TF), and target gene regulation and motif enrichment in the promoter regions. Moreover, we established the protein–protein interaction (PPI) networks for the genes that separated the cells into good and poor TKI responders. By overlaying a licensed anticancer drug database on the PPI networks, the drugs that directly or through PPI interactions indirectly targeted the gene markers of TKI response were uncovered. Our findings may lead to new therapeutic strategies for overcoming treatment resistance and relapse of CML.

## 2. Results

### 2.1. CP-CML Stem Cells

We obtained single-cell RNAseq data of stem cells from six healthy human donors and 16 patients with chronic-phase CML at diagnosis (Materials and Methods) [23]. After diagnosis, the patients were treated with TKIs. Based on the achievement of a major molecular response (MMR) to TKI, which is defined as a BCR-ABL transcript level of 0.1% or under, those patients were stratified into good responders (n = 11) and poor responders (n = 5). The BCR-ABL^+^ and BCR-ABL^−^ stem cells within each patient were separated and sequenced (Supplementary Figure S1). Low-quality cells were removed. A total of 232 normal hematopoietic stem cells (HSCs) and 762 CP-CML stem cells, composed of 255 BCR-ABL^+^ and 188 BCR-ABL^−^ cells from good responders and 181 BCR-ABL^+^ and 138 BCR-ABL^−^ cells from poor responders (Table 1), remained in the subsequent analysis. The patient ages ranged from 24 to 73 years in good responders and from 23 to 73 years old in poor responders. No significant association between age and TKI response was found (Supplementary Figure S2, Kolmogorov–Smirnov test *p* > 0.9).

### 2.2. Abnormal Gene Expression in Different Types of CP-CML Stem Cells

**Figure 1:**
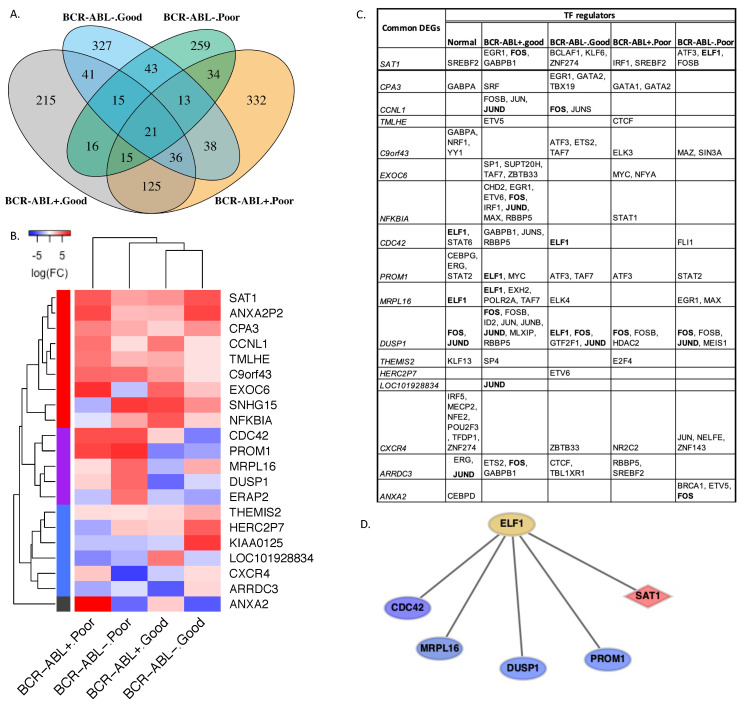
Gene expression and regulation changes in different CP-CML stem cells. (**A**) The pairwise comparisons between normal HSCs and different stem cell groups, which were BCR-ABL^+^ and BCR-ABL^−^ of good responders as well as BCR-ABL^+^ and BCR-ABL^−^ of poor responders, respectively, resulted in four differentially expressed gene sets. (**B**) The hierarchical clusters of the stem cell groups were based on expression changes of 21 common differentially expressed genes. The cells from the patients with the same TKI response (either good or poor) were clustered together. Meanwhile, the genes formed four clusters (red, purple, blue, and grey) according to their expression alteration patterns in different cell groups as compared to normal HSCs. (**C**) The TF regulators of common DEGs were identified from cellular transcription networks. The bold gene names in the table represent the most frequent TF regulators of common DEGs. (**D**) The transcription regulon of *ELF1*, one of the most frequent regulators in the transcription networks, controlled four underexpressed genes, shown in blue nodes, in the cells of good responders or normal HSCs, and one overexpressed gene, shown in the red node, in the cells of poor responders.

We studied gene expression changes in the four stem cell groups from CP-CML patients (Table 1) as compared to normal HSCs from healthy donors. Genes that were expressed in at least 10% of BCR-ABL^−^ cells and at least 20% of BCR-ABL^+^ cells for both good and poor responders were used in the pairwise differential analysis. As a result, 484 (out of 7992), 614 (out of 7843), 534 (out of 9889), and 416 (out of 9687) differentially expressed genes (DEGs) (|log(FC)|≥1 and p−adjust≤0.05) were identified in the stem cells of BCR-ABL^+^ from good responders, BCR-ABL^+^ from poor responders, BCR-ABL^−^ from good responders, and BCR-ABL^−^ from poor responders, respectively (Figure 1A). The DEGs of BCR-ABL^+^ cells were enriched in multiple pathways related to the immune system, such as the toll receptor signaling pathway and T cell activation. However, the DEGs in BCR-ABL^−^ stem cells were significantly enriched in pathways that control cell proliferation, cell differentiation, and cell apoptosis, such as the RAS pathway and P38 MAPK (mitogen-activated protein kinase) pathway. The deregulation of these genes in cancers often leads to increased invasion and metastasis and decreased apoptosis.

Among the four differentially expressed gene sets, good and poor responders shared 197 and 92 DEGs in the BCR-ABL^+^ and BCR-ABL^−^ stem cell groups, respectively. The gene set enrichment analysis showed that the common DEGs from good and poor responders in the BCR-ABL^+^ cells were significantly abundant in a number of biological processes including regulation of osteoblast differentiation (FDR < 0.03), transmembrane receptor protein tyrosine kinase signaling pathway (FDR < 0.02), regulation of immune system process (FDR < 0.03), and response to stress (FDR < 0.01). The significantly altered pathways included MAPK1/MAPK3 (mitogen-activated protein kinase 1/mitogen-activated protein kinase 3) (FDR < 0.008), RAF/MAP kinase cascade (FDR < 0.03), and signaling by VEGF (vascular endothelial growth factor) (FDR < 0.03). In contrast, according to FDR values, no significant associations between biological process, signal pathways, and common DEGs in the BCR-ABL^−^ cells of distinct TKI responders were found.

Furthermore, compared to normal HSCs, we found 21 genes that were concordantly differentially expressed across the four CP-CML stem cell groups (Figure 1A). The hierarchy clustering analysis according to the expression level changes of these genes with respect to the normal HSCs showed that the cells from the patients with the same TKI responses tend to cluster together (Figure 1B). Meanwhile, the DEGs were clustered into four sets (red, purple, blue, and grey, Figure 1B). The red gene set contained the genes upregulated in all cell types, the purple set included genes upregulated in the cells from poor responders while downregulated in the cells from good responders, and expression change patterns of genes in the blue and grey sets were more diverse across the four different cell groups. The three genes *CDC42* (cell division cycle 42), *PROM1* (prominin 1), and *DUSP1* (*dual specificity phosphatase 1*) in the purple gene set were associated with drug resistance in other types of cancer. The association between drug resistance and overexpression of *CDC42* was implicated in breast cancer cells [25]. *PROM1* was involved in regulations of drug resistance and metastasis in various cancer cells, and the expression and mutation of this gene were associated with poor prognosis in non-small lung cancer [26]. *DUSP1* was associated with drug resistance in multiple cancers [27,28,29]. Our results suggested these genes may also play essential roles in regulating TKI resistance in CML.

### 2.3. Inferring Cellular Regulatory Networks

We further applied SCENIC (single-cell regulatory network inference and clustering) to infer gene regulatory networks. A regression model was established based on the expression matrix of each cell group. The candidate regulatory modules composed of a TF and downstream target genes were built from gene co-expression patterns. The co-expression modules were further refined by eliminating the indirect target genes through TF motif enrichment analysis (see Methods for details). The refined transcription regulons for five stem cell groups including the four cell groups from CP-CML patients and normal HSCs were obtained.

Of the 21 common DEGs, 17 were found in the final refined gene regulatory networks (Figure 1C). The 17 DEGs were all target genes that were connected with one or more upstream TFs. *DUSP1*, *PROM1*, and *SAT1* (spermidine/spermine N1-acetyltransferase 1) had direct TF regulators in all cell groups, while *HERC2P7* (hect domain and RLD 2 pseudogene 7) and *LOC101928834* were regulated directly by TF only in the cells from good responders. Interestingly, the DEGs appeared to have more TF regulators in the cells from good responders than poor responders. The most frequent TF regulators, encoded by *FOS* (fos proto-oncogene, AP-1 transcription factor subunit), *ELF1* (E74-like ETS transcription factor 1), *JUND* (JunD proto-oncogene, AP-1 transcription factor subunit), and *TAF7* (TATA-box binding-protein-associated factor 7) (Figure 1C), controlled the expression of several DEGs in distinct cell groups. These frequent TF regulators participated in multiple signaling pathways including signaling by receptor tyrosine kinases (FDR = $4.48\times 10^{-2}$), signaling by nuclear receptors (FDR = $7.28\times 10^{-3}$), signaling by *NTRK1* (neurotrophic tyrosine kinase receptor) (FDR = $1.40\times 10^{-3}$), and nuclear events (kinase and transcription factor activation, FDR = $3.55\times 10^{-4}$). *ELF1*, encoding a member of ETS family transcription factors with vital roles in tumorigenesis, acts as a tumor suppressor [30]. *ELF1* mediated five common DEGs including four under-expressed (*CDC42*, *MPRL16*, *DUSP1*, and *PROM1*) genes in cells of good responders, and one over-expressed (*SAT1*) gene in the cells of poor responders (Figure 1D).

### 2.4. CP-CML Stem Cells with Different TKI Response

**Figure 2:**
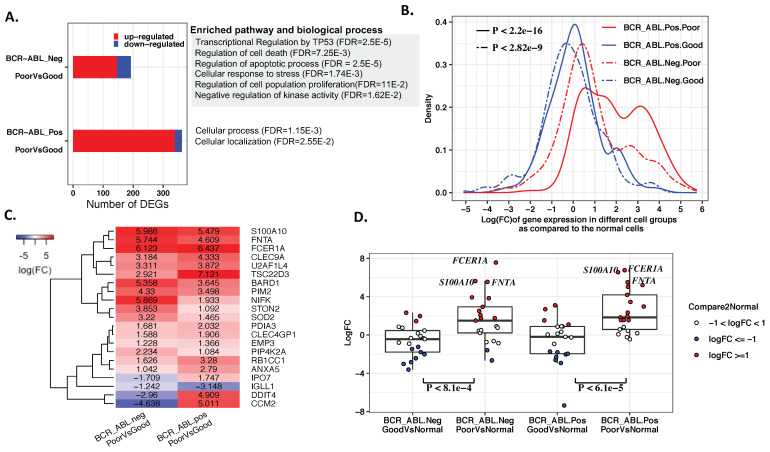
Comparisons of the stem cells from distinct TKI responders. (**A**) The gene expression markers that differentiate the cells of good and poor responders in BCR-ABL^−^ and BCR-ABL^+^ cell groups. Most gene markers were upregulated in the cells of poor responders. TKI response markers were enriched in multiple biological processes and pathways related to cancer development and drug resistance for the BCR-ABL^−^ cell group, while similar function disruptions were not observed in BCR-ABL^+^ stem cells. (**B**) The distribution of expression changes of the response gene markers in the individual cell groups as compared with the normal HSCs. The red curves correspond to expression change in the cells of the poor responders, while the blue curves are for good responders. (**C**) The major TKI response marker genes shared by BCR-ABL^−^ and BCR-ABL^+^ stem cells had higher expression in the cells of poor responders than good responders. (**D**) The expression changes of the common gene markers as compared to the normal HSCs. *S100A10*, *FCER1A*, and *FNTA* had the largest expression increase in the cells of TKI poor responders. These genes showed more elevated expression levels in the both BCR-ABL^+^ (*p* < 6.1 × $10^{-5}$) and BCR-ABL^−^ (*p* < $8.1\times 10^{-4}$) stem cells from poor responders than from good responders.

To further focus on studying gene alterations in the cells from distinct TKI responders, we conducted differential gene expression analysis between the stem cells from the patients with good and poor responses in the BCR-ABL^+^ and BCR-ABL^−^ clusters, respectively. A total of 10411 and 9639 genes that were expressed in at least 10% of BCR-ABL^+^ and BCR-ABL^−^ from both good and poor responders were used in the differential expression analysis. As a result, 362 and 192 differentially expressed genes (abs(logFC) ≥ 1 and padj ≤ 0.05) were identified in the BCR-ABL^+^ and BCR-ABL^−^ cell groups. Among these genes, 93.6% (339/362) and 76% (146/192) were upregulated in the cells of poor responders compared to the cells of good responders in the BCR-ABL^+^ group and BCR-ABL^−^ group, respectively (Figure 2A). When compared to normal HSCs, the expression change distributions of these genes were significantly different between the patients with good and poor responses for BCR-ABL^+^ (Kolmogorov–Smirnov test, *p* < 2.2 × $10^{-16}$ Figure 2B, solid red vs. blue curves) and BCR-ABL^−^ stem cells (Kolmogorov–Smirnov test, *p* < 2.82 × $10^{-9}$, Figure 2B, dashed red vs. blue curves). The majority of these genes had elevated expression levels in the cells of poor responders than their expression in the normal HSCs in both BCR-ABL^+^ and BCR-ABL^−^ cells (Figure 2B, red curves).

The genes that differentiated TKI responders in the BCR-ABL^−^ stem cells were enriched in transcriptional regulation by the P53 (FDR = $7.67\times 10^{-3}$) pathway and prevalent in a number of biological processes related to cancer including regulation of cell death (FDR = $7.25\times 10^{-6}$), regulation of apoptotic process (FDR = $2.50\times 10^{-5}$), cellular response to stress (FDR = $1.74\times 10^{-3}$), regulation of cell population proliferation (FDR = 1.10 × $10^{-2}$), and negative regulation of kinase activity (FDR = 1.62 × $10^{-2}$). In contrast, DEGs in the BCR-ABL^+^ cells only revealed two general biological process terms including cellular process (FDR = 1.15 × $10^{-3}$) and cellular localization (FDR = 2.55 × $10^{-2}$), and no enriched pathway was found at FDR ≤ 0.05. Distinct enriched biological process terms and pathways suggested different mechanisms underlying cell TKI response for the BCR-ABL^+^ and BCR-ABL^−^ cells.

On the other hand, we found that 21 genes were concordantly differentially expressed between cells from different responders in both BCR-ABL^+^ and BCR-ABL^−^ stem cells (Figure 2C). These genes can be putative expression markers for predicting TKI responses. Seventeen of them were upregulated in the poor responders’ cells. Moreover, respective to gene expression levels in normal HSCs, we found that most of these genes had significantly higher expression levels in both BCR-ABL^+^ (Wilcox test, *p* < 6.1 × $10^{-5}$) and BCR-ABL^−^ (Wilcox test, *p* < 8.1 × $10^{-4}$) stem cells from poor responders than from good responders (Figure 2D). Collectively, our results suggested that elevated expression levels of essential genes were associated with poor TKI response. Three genes (Figure 2C,D), *S100A10* (S100 calcium-binding protein A10), *FCER1A* (Fc epsilon receptor Ia), and *FNTA* (farnesyltransferase, CAAX box, alpha), displayed the largest increased expression levels in the TKI poor responders in both comparisons with the cells of good responders and with the normal HSCs. *S100A10* is correlated with drug resistance to various cancer types [31,32,33,34]. On the opposite side, the roles of *FCER1A* and *FNTA* in cancer and therapeutic resistance have not been well studied and thus could be novel markers of therapeutic resistance in CML.

### 2.5. Protein–Protein Interaction Networks Reveal Putative Drugs for Response Predictive Markers

We hypothesized that the gene markers that differentiated cells from the patients with distinct TKI responses can be used for predicting the treatment response as well as be considered as new putative drug targets for improving CML therapeutic efficacy. We searched an anticancer drug database [35] to explore whether any existing drugs are available for these genes. Four gene markers, *BARD1* (BRCA-associated RING domain 1), *SOD2* (superoxide dismutase 2), *S100A10*, and *TSC22D3* (TSC22 domain family member 3), were found to be the targets of several approved cancer drugs. *BARD1* encodes a protein that regulates tumor suppression and cell growth [36], and interacts with the N-terminal region of *BRCA1* (breast cancer gene 1). Mutation of *BARD1* was implicated in multiple types of cancer. *SOD2* regulates mitochondrial superoxide scavenger and can either suppress or promote tumor growth [37]. *S100A10* is responsible for nearly 50% of cellular plasmin generation and controls cancer cell invasion and metastasis as well as recruits tumor-associated cells to the tumor site [38]. *TSC22D3* has a role in anti-inflammatory and immunosuppressive effects [39]. *S100A10* and *TSC22D3* are the targets of the same anticancer drug dexamethasone (Supplementary Table S1), while both *BARD1* and *SOD2* have five anticancer drugs.

**Figure 3:**
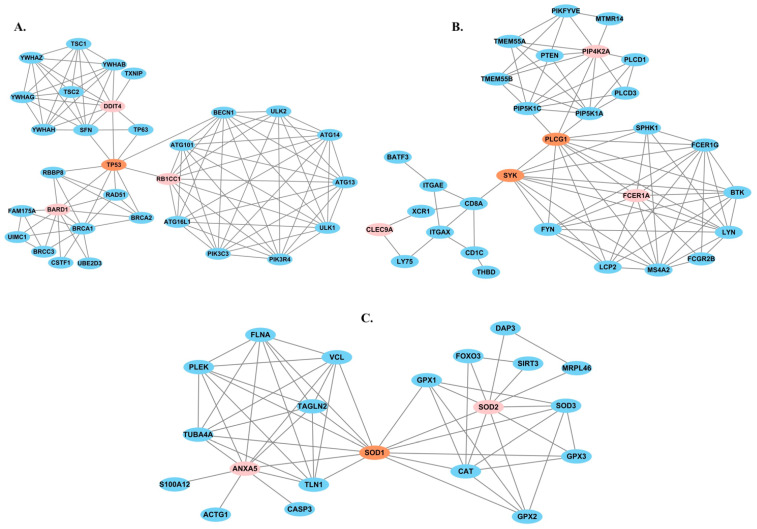
The PPI networks of response predictive gene markers. (**A**) *TP53* connected the PPI networks of *BARD1*, *RB1CC1*, and *DDIT4*. (**B**) *PLCG1* and *SYK* connected the PPI networks of *FCER1A*, *PIP4k2A*, and *CLEC9A*. (**C**) *SOD1* connected the PPI networks of *SOD2* and *ANXA5*.

We expanded the analysis by establishing protein interaction networks for the gene markers of TKI response based on STRING human protein–protein interactions (PPIs) with multiple layers of experimental evidence (Materials and Methods). The protein interaction networks of the twenty markers, except for *CLEC4GP1* (C-type lectin domain family 4 member G pseudogene 1), were identified (Supplementary Table S2). Via *TP53*, *PLCG1* (phospholipase C gamma 1), *SYK* (spleen-associated tyrosine kinase), and *SOD1* (superoxide dismutase 1), protein–protein interaction networks of nine predictive markers were interconnected (Figure 3). *TP53* encodes p53 tumor suppressor protein and is the most frequently mutated gene in cancer. Two anticancer drugs, trifluridine and tipiracil, target *TP53* [40]. *PLCG1* is a member of phospholipase C (PLC) family, which may be changed by kinase activity in the cancer progression. *PLCG1* can activate cellular proliferation in response to growth factors such as *EGFR* (epidermal growth factor receptor) [41]. *SYK* is a kinase inhibitor therapeutic target for acute myeloid leukemia [42]. *SOD1* is associated with cisplatin resistance [43]. Taken together, the known roles of *TP53*, *PLCG1*, *SYK*, and *SOD1* and their interactions with multiple PPI networks of gene expression markers suggested their roles in the mediating distinct cell response to TKI treatment of CML. We further overlaid cancer drugs and target genes with the PPI networks and found that additional 14 mark gene protein products interacted with the target genes of existing cancer drugs. Most of these drugs demonstrated anticancer effects in cancer types other than CML. Our findings indicated they could be further examined and repurposed for deeper cellular response to TKI treatment for CML patients.

## 3. Materials and Methods

### 3.1. Data Process and Visualization

The single-cell RNA-seq data (GSE76312) of human CML and normal hematopoietic stem cells were downloaded from Gene Expression Omnibus (GEO) [44]. A highly sensitive BCR-ABL detection single-cell sequencing protocol was utilized for sequencing the transcriptomes of BCR-ABL^+^ and BCR-ABL^−^ stem cells from the individual CML patients [23]. The protocol can increase the BCR-ABL detection rate to 100% in K562 cells for microfluidic-based or plate-based platforms [23]. Gene expression values were quantified as read per kilobase of transcript length per million mapped reads (RPKM) based on the RefSeq gene model using the *rpkmforgenes* [45]. We selected genes expressed in more than ten cells with a coefficient variation score (standard deviation/mean) ≥ 1. Then, expression values were converted to log2(RPKM) for the following analysis. Genes with the sum of log2(RPKM) of all analyzed cells less than 1 were removed. When the value of RPKM < 1, its log-transferred value was set to 0.

The Wilcox test and Fisher exact test were employed for differential expression analysis. The genes that achieved |log(FC)| ≥ 1, p−adjust≤ 0.05, and were expressed in at least 10% of the examined cell groups were considered as differentially expressed. PANTHER [46] was used for gene ontology and pathway enrichment analysis. We used the R package *tsne* to visualize cell clusters in two-dimension space. t-stochastic neighbor embedding (t-SNE) is a non-linear dimensionality reduction approach and it can map high-dimensional datasets into a space of two or three dimensions.

### 3.2. Transcription Network Inference

SCENIC [47] was applied to infer the transcription network. The input files of SCENIC included scRNA-seq expression matrix and a list of human transcript factors (TFs) including 1839 genes [47]. First, a regression model, GRNBoost2 [48], was applied to predict the expression of a gene across cells based on the expression of the TFs. The output of this step was a list of pairwise connections between the TF and a target gene, and a weight of the connection measuring the strength of regulatory interactions. Next, the regulatory pairs with strong regulatory relations were merged into modules for each of the TFs. The positively correlated modules were retained for the subsequent step. Additionally, the modules with less than twenty genes were removed.

The modules contained direct and indirect targets of a TF regulator since the regulatory interactions were established based on co-expression patterns. Then, the target genes were further refined by searching for the direct TF binding sites of the gene. The promoter region between the downstream 100 base pairs and upstream 500 base pairs of the transcription start site of a target gene was extracted and searched for motif enrichment. Here, human genome hg38 assembly was used. If the motif of the TF was enriched in one of its modules, the TF and its predicted targets were kept for further analysis. The motif enrichment analysis also determined a leading edge and thereby prune the target genes of a module. Finally, the set of predicted direct target genes across all modules sharing the same regulator was combined into one regulon.

### 3.3. Protein Interaction Network

The PPIs in the STRING database [49] are supported by multiple levels of evidence such as homology, co-expression, experimentally determined interactions, database annotated, and automated text mining [50]. Each piece of evidence is associated with a score between 0 and 1. The larger score, the higher level of confidence. We employed three criteria including experimentally determined interaction, database annotation, and automated text mining to select interactions. An interaction that has a minimum score of 0.8 in one or more of the three criteria was utilized to construct PPI networks of the gene markers.

### 3.4. Cancer Drug Database

The drug database [35] consists of 285 licensed anticancer drugs that meet certain criteria. First, the drug is utilized for anticancer effects rather than for diagnosis, supportive care, or treating cancer-related morbidities; secondly, the drug is approved for treating one or more cancer malignancies by at least one regulatory agency such as the FDA or EMA. Approximately 90% and 62% of drugs in the databases were approved by FDA and EMA, and about 19% were approved by various European countries.

## 4. Discussion

The protein product of the BCR-ABL fusion gene causes uncontrolled CML cell growth. TKI targets this oncoprotein and inhibits protein kinase activity to eliminate CML cells. TKI therapy has substantially improved the outcome of CML patients. However, the cure rate remains low. Some CML patients either experience inadequate initial responses, lose response to the treatment, or suffer from tolerability issues [51]. Additionally, treatment discontinuation can result in relapse [52]. Intra- and inter-tumor heterogeneity is a general characteristic of many cancer types including CML and is considered one of the major causes of drug resistance and treatment failure. Thus, it is essential to investigate various cell subpopulations for dissecting diverse mechanisms driving drug response. Unlike traditional batch RNA sequencing and microarray, which measure the average expression level of tens of thousands of cells, single-cell RNAseq offers high-resolution gene expression measurement at the single-cell level. Hence, the single-cell-based analysis enables new treatment strategies to be developed.

In this study, we analyzed the scRNA-seq expression profiles of BCR-ABL^+^ and BCR-ABL^−^ stem cells from individual CP-CML patients. Our results showed that the enriched biological process and pathways of differentially expressed genes between normal HSCs and each of the CP-CML stem cell groups were diverse. Multiple cancer-related biological processes and signal pathways were abundant in the DEGs shared by BCR-ABL^+^ stem cells from both good and poor TKI responders, whereas no biological processes or pathways were significantly enriched in the common DEGs in BCR-ABL^−^ stem cells in the good and poor responders. Consistent with this observation, the t-SNE plots using the common DEGs demonstrated that the cells with different response categories were not separated well in the BCR-ABL^+^ stem cells, by contrast, they formed two clear clusters in the BCR-ABL^−^ stem cells (Supplementary Figure S3). On the other hand, the genes that discriminate between good and poor response groups were not enrichment in any biological process and pathway in BCR-ABL^+^ cells, while multiple diseases and drug resistance related to biological processes and pathways were abundant in the gene markers of TKI response in the BCR-ABL^−^ cells. Taken together, our findings suggested that distinct mechanisms underlie disruptions in the BCR-ABL^+^ and BCR-ABL^−^ stem cells and the disruptions of BCR-ABL^−^ stem cells of CML patients are associated with TKI response. A better understanding of both stem cell types can help us to improve current treatment and relapse prevention.

The three genes *S100A10*, *FCER1A*, and *FNTA* had the largest expression alterations compared to normal HSCs as well as in the comparison between good and poor responders (Figure 2C,D). The product of *S100A10* is a member of the S100 protein family and is involved in Ca^2^+ and Mg^2^+ transport [53,54]. It has been reported that *S100A10* plays a key regulatory role of toll-like receptors that can activate the innate immune system and are associated with tumor growth [55]. The abnormal expression of *S100A10* was associated with drug resistance in multiple cancer types including colorectal cancer [31], neuroblastoma [32], breast cancer [33], and ovarian cancer [34]. *FCER1A*, an innate immunity gene [56], binds to the Fc region of immunoglobulins epsilon and is responsible for initiating the allergic response and inducing the secretion of essential lymphokines. The mutations of *FCER1A* have been reported in multiple human allergic disease studies [57,58,59]. Lee et al. [60] reported the relationship between *FCER1A* mutation and breast cancer risk and reasoned that the immune-stimulating conditions caused by the mutations may contribute to susceptibility to breast cancer. *FNTA* is involved in the programmed cell death pathway. Jiang et al. knocked down *FNTA* and *RabGGTA* genes to block functions of *Ras* and *Rab* [61]. *Ras* and *Rab* belong to the RAS superfamily, which has important roles in regulating cellular processes and signal transduction such as proliferation, differentiation [62]. Compared to *S100A10*, presently much fewer studies explore the relations between *FCER1A*, *FNTA*, and cancer.

We identified the candidate drugs that target gene markers of TKI response or their interacting proteins in both BCR-ABL^+^ and BCR-ABL^−^ stem cells. For example, *S100A10* and *TSC22D3A* were upregulated in the cells of poor responders. The anticancer drug dexamethasone targets both genes. This drug has been used for acute leukemia, malignant lymphomas, multiple myeloma, and mycosis fungoides. Our analysis indicated that the usage of dexamethasone in addition to the first-line drug in CML treatment might potentially enhance the therapy efficacy. Nevertheless, our work enables further study of the existing drug effects in the cellular network context based on single-cell RNAseq data analysis, which may lead to identifying optimal therapy for CML.

The mean age of diagnosis of CML is approximately 64 years [63]. However, the disease can occur in all age groups. Pemmaraju et al. investigated outcomes in adolescents and young adults (n = 61), defined as those aged 15-20 years, compared to their older counterparts (age ≥ 30, n = 407). Based on a multivariate analysis of 13 factors such as percentage of Philadelphia chromosome metaphases, age group, and Sokal risk score, they found that adolescents and young adults were associated with a low probability of achieving complete cytogenetic response (84% vs. 93%) and major molecular response (75% vs. 86%) [64]. They argued that the unfavorable outcomes for young patients may be related to both biological and non-biological features such as psychosocial elements, adherence to therapy, and access to health care and medical insurance. In our analysis, no statistically significant association between TKI response and age was detected. This may result from the small size of the patient cohort, which consisted of 2 patients younger than 30 years and 14 patients older than 30 years. Nevertheless, the distinct MMR of the CML patients in this work was most likely caused by molecular and cellular differences rather than age. Future age group-specific single-cell sequencing and downstream analysis may help to develop age-adapted treatment strategies.

## 5. Conclusions

In this study, we integrated analyses of single-cell RNAseq data of CP-CML stem cells and network inference to characterize intratumor heterogeneity and therapy responses in CML. Group-specific and common differentially expressed genes were identified across four CML stem cell groups. The subsequent hierarchy clustering analysis demonstrated that the cell groups with the same molecular TKI responses were clustered together. Gene regulatory network analysis uncovered the TF regulators of these genes, which can help us to better understand the cellular regulatory mechanisms underlying therapy response. Additionally, we found the majority of gene markers were upregulated in the stem cells of patients with poor response. Moreover, the drugs that target these marker genes may be repurposed for improving CML treatment.

## Figures and Tables

**Table 1:** Stem cell groups from CP-CML patients.

BCR-ABL	TKI Response	
	Good	Poor
**Positive**	255	181
**Negative**	188	138

## Data Availability

Cancer drugs interconnect with the TKI response gene markers Supplementary Table S1), and protein interaction subnetworks of the TKI responses gene markers (Supplementary Table S2).

## Supplementary Materials

The following supporting information can be downloaded at: https://www.mdpi.com/article/10.3390/ijms232214335/s1.
