# Supplementary figures and images for: Integrating Single-Cell Transcriptome and Network Analysis to Characterize the Therapeutic Response of Chronic Myeloid Leukemia
Source: Int J Mol Sci. 2022 Nov 18;23(22):14335. doi: 10.3390/ijms232214335 (PMC9695508; doi:10.3390/ijms232214335)

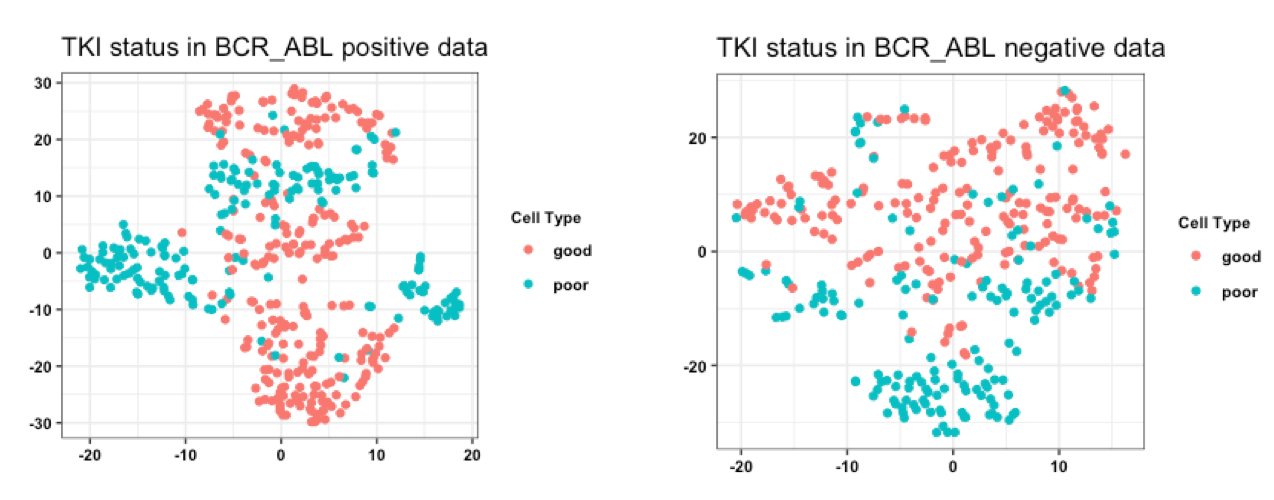

Supplement: Supplementary file 1 [file ijms-23-14335-s001.zip › Figur S3.png]

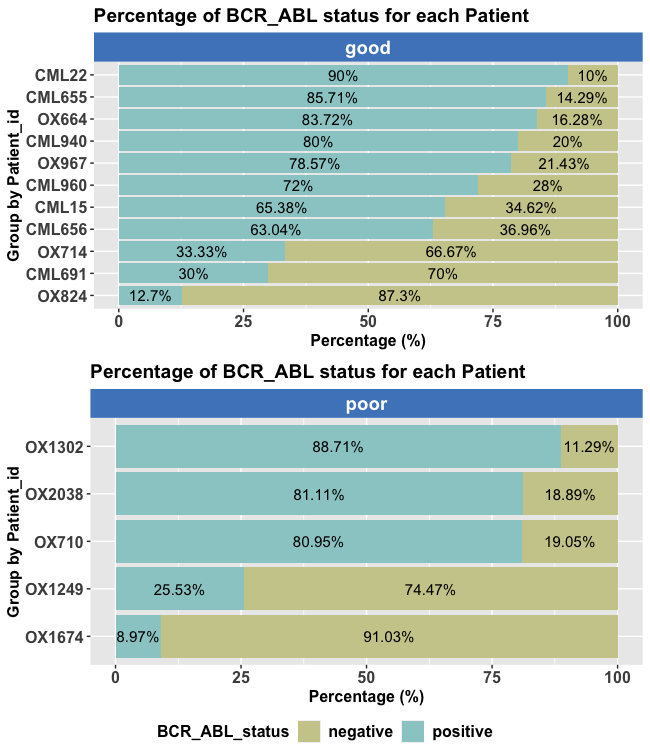

Supplement: Supplementary file 1 [file ijms-23-14335-s001.zip › Figure S1.png]

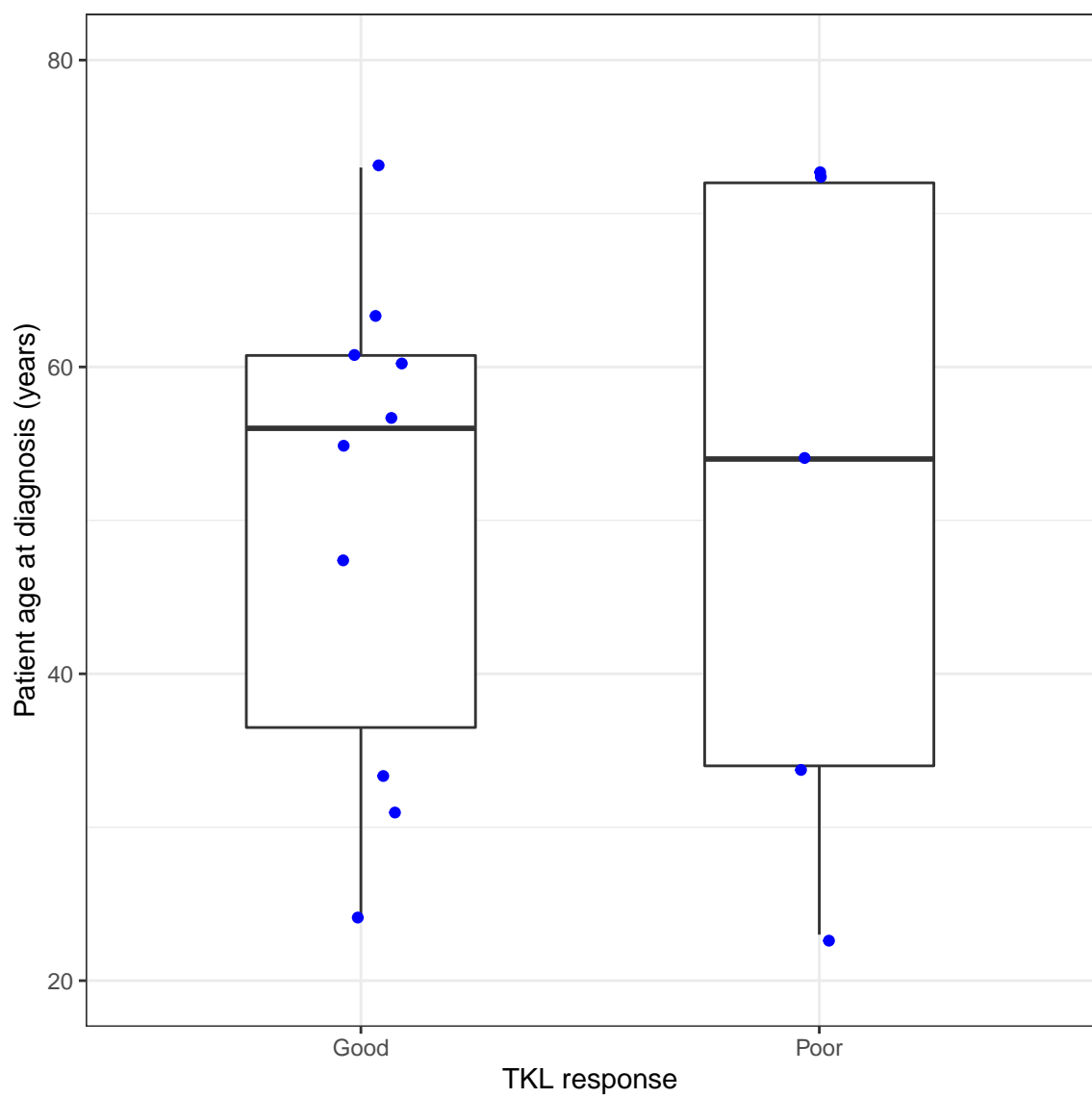

Supplement: Supplementary file 1 [file ijms-23-14335-s001.zip › Figure S2.pdf]
